# Determination of spider mite abundance in soil of field-grown cucumbers and in plants under predatory mite pressure in invasive infestations using HRM real-time PCR assay

**DOI:** 10.1371/journal.pone.0270068

**Published:** 2022-07-14

**Authors:** Anne-Katrin Kersten, Carmen Büttner, Peter Lentzsch

**Affiliations:** 1 Research Area 1 “Landscape Functioning”, Leibniz Centre for Agricultural Landscape Research (ZALF), Müncheberg, Germany; 2 Faculty of Life Science, Thaer-Institute of Agricultural and Horticultural Sciences, Division Phytomedicine, Humboldt-Universität zu Berlin, Berlin, Germany; Universidade Federal de Lavras, BRAZIL

## Abstract

The two spotted spider mite, *Tetranychus urticae* Koch L. (Acari: Tetranychidae), is a plant pest that can lead to severe economic losses in open field cucumber cultivation. Between 2017 and 2019 we studied the abundance of spider mites in the soil to estimate the potential infestation pressure of soil colonizing spider mites. The spider mites were heterogeneously distributed in small concentrations in the soil. Soil colonizing spider mites did not affect spider mite abundance on plants and reversed. We observed that spider mite migration occurred primarily from the edge of the field adjacent to the weed strip. In 2020 and 2021, we investigated the efficacy of the predatory mite *Neoseiulus californicus* (McGregor) for suppressing spider mite hotspots in the cropland. We compared untreated spider mite hotspots with *N*. *californicus* treated hotspots and showed that a single release of predatory mites could result in a high level of control when spider mite infestation density was initially high. With this study, soil can be ruled out as a habitat for spider mites, and attention to spider mite pest control can be directed to plant infestations. The highly sensitive HRM real-time PCR assay was used for the quantification of the spider mites.

## Introduction

The Two-Spotted spider mite, *Tetranychus urticae* Koch, (Acari: Tetranychidae), is a globally distributed phytophagous and economically important pest that attack over 1,169 plant hosts, some with economic importance. Wheat, peanut, cotton and ornamentals, as well as vegetables such as pepper, tomato and cucumber are infested by spider mites [1–4].

Cucumbers (*Cucumis sativus* L.) are often exposed to a serious infestation of spider mites and could be attacked by 10 to 15 generations of this pest per year under temperate climate conditions [5]. A single spider mite can cause a yield loss of 5.03 g per cucumber plant in a greenhouse cultivation just in spring [6]. This enormous harmful effect can lead to high yield losses in commercial cultivation and needs to be counteracted, especially since cucumber ranks among one of the widely cultivated cucurbits worldwide [7].

The use of acaricides and insecticides is currently the most common control method of *T*. *urticae* and related *Tetranychus* species [8]. However, a problem in effective spider mite control is the long-term use of those chemical agents and the rapid development of resistance due to the high reproduction rate and short generation time of spider mites. Furthermore, modern agriculture with monoculture and the use of pesticides eliminates natural coexistence with predators and creates conditions favorable for spider mites to grow in high density [9]. Predatory mite release of the family *Phytoseiidae* is a well-known control agent of *T*. *urticae* for greenhouse cultivation [10, 11] but has not been well documented in the field. The control efficiency of these commercially used predatory mites decreases under dry and hot conditions while these increasingly common conditions lead to an explosive proliferation and spread of spider mites, which have a development period of 10.26 days on cucumbers at 35°C under laboratory conditions [5]. The predatory mite *Neoseiulus californicus* (McGregor) has been proven to be more resilient to variable temperatures and humidity, as well as prey deficiency, than the commonly used *Phytoseiulus persimilis* Athias-Henriot [12–14]. Biological control agents such as the application of predatory mites are popular, but curative releases of predatory mites like *N*. *californicus* every two to four weeks as recommended is approximately five times the cost of the chemical alternative [15, 16]. Targeted and localized release of predatory mites could be a more cost-effective application method.

*T*. *urticae* infests new host plants by crawling or wind assisted when the quality of the host declines due to mite-induced injuries, plant senescence, or harvest [17, 18]. Therefore, *T*. *urticae* exploits diverse habitats throughout the year [18, 19]. Knowing the different habitats or potential sources of spider mites is mandatory for spider mite management and for the targeted use of control agents. Previous studies showed that weeds, litter, clods of soil, cracks in trees and barks, or plant debris serve as (overwintering) shelters for spider mites [19, 20] and that spider mites prefer dark shelters for overwintering [21]. Moreover, it is well studied that spider mites migrate mainly from the surrounding weed edges into the cropland [17]. Studies on the soil as potential shelter and source of spider mite infestation in open field cucumber cultivation are lacking.

The aim of our study was to gain knowledge about the abundance of *Tetranychus* spider mites in the soil of cucumber cultivation in order to assess the soil as a potential habitat by spider mites. The goal was to understand the importance of soil as a source of spider mite infestation in cucumber production and to determine how intensive soil needs to be involved in control strategies, especially in invasive infestations. In addition, we considered the effect of a targeted one-time release of *N*. *califonicus* in spider mite hotspots, thus limiting the application of predators to a small area and reducing costs. The study emphasized practical relevance in order to be able to apply the knowledge gained and strategies developed here.

## Materials and methods

### Open field plot design

**Fig 1:**
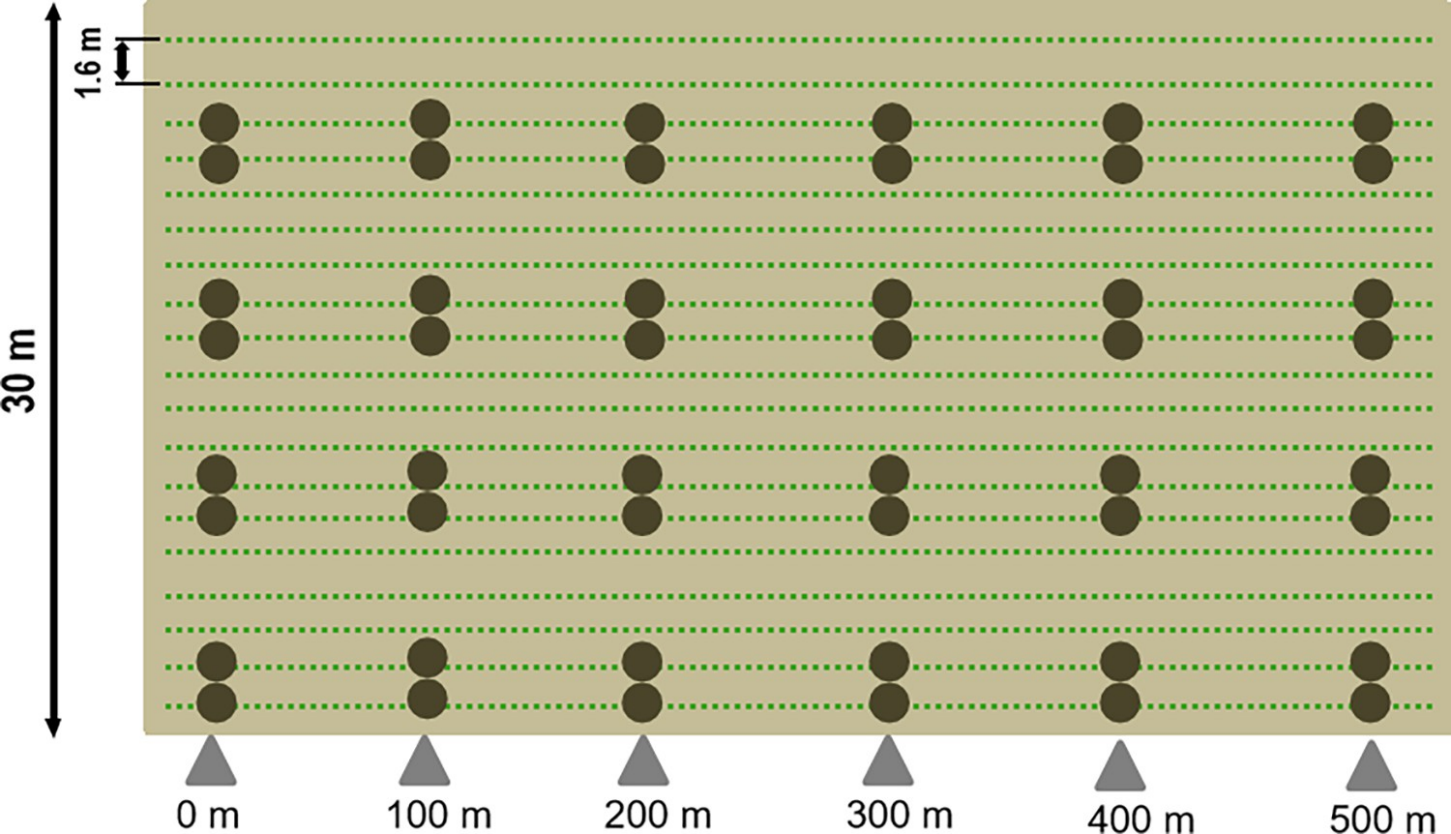
Schematic design of an exemplary trial plot. A total of nine trial plots of different sites in the Spreewald region were analysed in 2017, 2018 and 2019. All trial plots were analysed according to the sampling design shown here. Trial plot dimensions were 500 m long and 30 m wide with 17 to 19 rows of planted cucumbers (green dotted line) included. Transects with GPS-coded sample points (brown dots) were established every 100 m.

The trial plots were integrated into commercially farmed open field cucumber cropland located in the Spreewald region, in eastern Germany. A total of 12 different cultivation areas with the same subsoil irrigation and fertilization were analysed between 2017 and 2021 (different plots were analysed each year; no duplication of trial plots). The defined trial plots were 500 m long and 30 m wide with 17 to 19 rows of cucumber plants of the cultivars ‘Liszt‘ or ‘Platina‘ (Fig 1). Two trial plots were always placed side by side and combined to one plot, resulting in an analysed area of 30,000 m^2^. Due to the harvesting method of cucumbers, there was a 2.5 m wide rut for the harvester between the two trial plots. Distances between plants (30 cm) and plant rows (160 cm) were standardized and adapted to the harvesters. Transects with GPS-coded sample points were defined every 100 m in each trial plot. The sample points were placed on plant rows and maintained during growing season (Fig 1). Samples from two adjacent sample points were combined into one mixed sample. There were a total of eight regularly spaced sampling points on a transect, resulting in 48 mixed soil samples on one plot.

### Sampling 2017 to 2019

Three trial plots of different sites in the Spreewald region were analysed in each of the years 2017 (plot A; B; C), 2018 (plot D; E; F) and 2019 (plot G; H; I) (no duplication of the trial plots). Soil samples were collected per sampling point to a depth of 20 cm with a core volume of 56.55 cm^3^ using a drill rod (diameter 2 cm). In total 48 mixed samples of soil were analysed per plot (Fig 1, brown dots). Soil samples were collected before planting the cucumbers (April), during the main harvest (end of July) and after the end of the harvest (September) each year. In 2018, additional sampling of soil and foliage (was conducted at plot D (over a 30 m width)) at 24 sample points in mid-June. Three to Five leaves and two soil samples were collected at one sampling point and merged together for analysis. Leaves of approximately the same age and 30 cm from the shoot tip were taken to ensure that the leaves were sampled as homogeneously as possible at the various sampling sites. Due to severe leaf damage caused by pest infestation, the collected leaves weighed approximately 2 g at a size of 10x10 cm. The degree of spider mite density was measured in spider mite number/ g leaf by HRM (high-resolution melt analysis) real-time PCR. All samples were sterile packed, transported refrigerated and stored at 4°C (soil) or -20°C (foliage) until further analysis after 10 to 20 days.

### Sampling of spider mite hotspots and predatory mite release in 2020 and 2021

**Fig 2:**
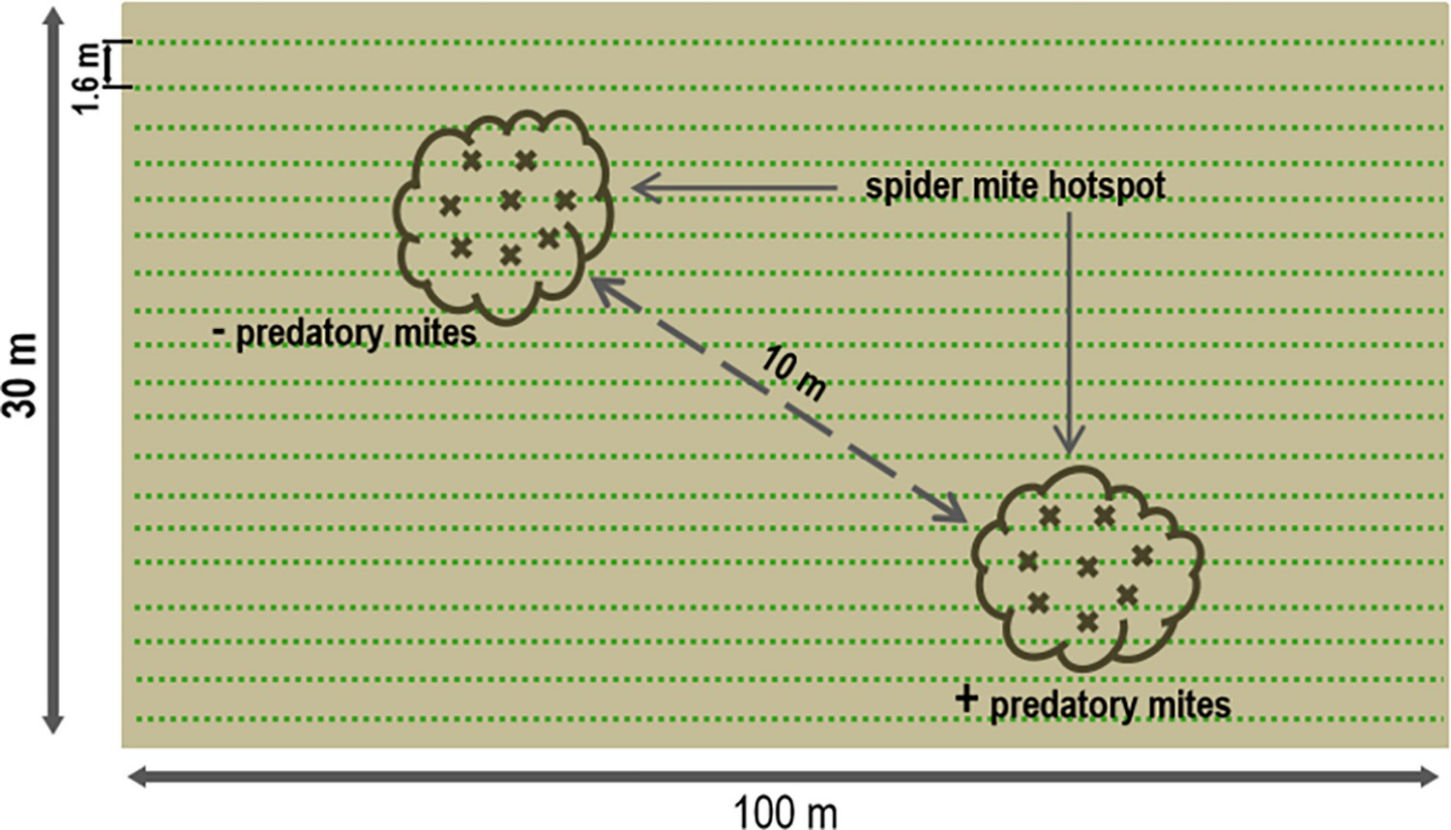
Schematic representation of spider mite hotspots in a trial plot. Visually detectable spider mite hotspots were analysed 2020 and 2021. Two spider mite hotspots were sampled per trial plot. One hotspot was treated with predatory mites (*Neoseiulus californicus* (Acari: Phytoseiidae) (McGregor)) (+ predatory mites), the second remained untreated and served as a control (- predatory mites). Hotspots had a minimum spacing of 10 m and a diameter of approximately 6 m. Crosses indicate sampling points for soil and foliage.

Visually detectable spider mite hotspots occurred within the trial plots (Fig 2) at different sites during the main cucumber harvest in the years 2020 (plot J; K) and 2021 (plot L) (no duplication of trial plots between the trial years).

A hotspot showed a clearly visible characteristic damage pattern by spider mites such as punctuate brightening on the leaves on 80% of the plants. Two spider mite hotspots were selected per trial plot, each with an approximate diameter of 6 m and a minimum spacing of 10 m apart. One of the two hotspots per trial plot served as untreated control (- predatory mites), whereas the second was treated with predatory mites (*N*. *californicus* (Acari: Phytoseiidae)) (+ predatory mites) (Katz Biotech AG, Baruth/Mark, Germany). The control hotspots (- predatory mites) were therefore also colonized with spider mites and represented the natural infestation process without countermeasures. Thus, spider mite dynamics could be compared in a total of three control (- predatory mites) and three treated hotspots (+ predatory mites). Randomly collected soil (4 to 8 samples, each 56.55 cm^3^) and foliage samples (8 to 12 leaves per hotspot, approximately 2 g each) were analysed weekly per hotspot for three weeks in late July to early August (first sampling date: T0; second sampling date: T1; third sampling date: T2). The samples collected at time T0 were used to determine the initial spider mite density. Samples taken at T1 and T2 were used to determine spider mite dynamics and to measure a potential predatory mite effect. The predatory mite release occurred once between T0 and T1 in the “treated”hotspots (+ predatory mites) in 2020 and 2021. Approximately 10,000 predatory mites were distributed as leaf material in a hotspot (+ predatory mites). According to the supplier, the commercially available leaf material includes predatory mites of all life stages (adult females ready to oviposit, nymphs, and eggs), and in case of infestation, up to 5 to 10 predatory mites/ m^2^ should be applied. This research on spider mite control by predatory mites should be close to practical application and not be limited to laboratory conditions. Therefore, spider mites detected were naturally occurring and not artificially exposed spider mites. The degree of spider mite density was measured in spider mite number/ g leaf by real-time PCR. All samples were sterile packed, transported refrigerated and stored at 4°C (soil) or -20°C (foliage) until further analysis after 10 to 20 days.

### DNA extraction

Spider mite abundance was determined via extraction of total DNA from collected samples (leaves or soil) followed by specific HRM real-time PCR. Two pooled soil samples were thoroughly mixed. Total DNA was extracted from 500 mg ± 1 mg soil using the NucleoSpin® Soil Kit (Macherey-Nagel GmbH and Co. KG, Düren, Germany; User’s Manual May 2016, Rev. 06) according to the manufacturer’s instructions. Soil samples for analysis were treated with FastPrep®-24 (M.P. Biomedicals, CA, USA) at 5 m/ s for 30 sec for mechanical lysis. Randomized parts of the collected leaves (3 to 5 leaves) from one sampling point were pooled and ground with 90 mg fine-grained silica sand in a mortar before DNA extraction. 400 to 700 mg of this homogenate were used for DNA extraction and therefore incubated in lysis buffer (Macherey-Nagel GmbH and Co. KG, Düren, Germany) at 22°C for 90 min in a rotary shaker (Enviro-Genie, Scientific Industries, Inc., NY, USA) and DNA was finally extracted according to the standard protocol of NucleoSpin® Soil Kit. Total amounts of purified DNA were assessed using NanoDrop ND-1000 (Kisker Biotech GmbH and Co. KG, Steinfurt, Germany) and stored at 4°C until further analysis.

### HRM real-time PCR assay

The *T*. *urticae* real-time PCR assay according to Li et al. (2015) [22] was conducted in a modified form using Quantstudio 12K Flex Real-Time-PCR-System (ThermoFisher Scientific, MA, USA). The specific primer Turti_1F: 5′-GTTTTACACTTCTTCGCCTAA-3′ (forward primer) and Turti-1R: 5′-CACCGCTTGAAGATGTATCT-3′ (reverse primer) are focused on the ITS1 region, with single nucleotide polymorphisms and indels present in non-targeted *Tetranychus* spp. sequences in the forward primer. Li et al. (2015) note that their probe real-time PCR assay is highly specific for *T*. *urticae* and is unlikely to be false positive in other unrelated species [22]. PCR reaction of 20 μL volume contained 4 μL 5x HOT FIREPol® EvaGreen®HRM Mix (solis Biodyne, Tartu, Estonia), 300 nM of each primer, and 1 μl DNA extracted from the sample. The cycling conditions include preincubation at 95°C for 15 min and an amplification program of 45 cycles as follows: denaturation 95°C, 15 s; annealing/ extension at 60°C for 20 s and finally high-resolution melt analysis (HRM) at 95°C for 15 s, 60°C for 60 s and 95°C for 15 s. The HRM real-time PCR method allows differentiation of genotypes [23]. The standard curve was carried along with each PCR run and based on extracted DNA from counted spider mites (*T*. *urticae*, Katz Biotech AG, Baruth/ Mark, Germany) in twofold execution. Based on the PCR run design, eight no template controls (NTC) were included in each PCR run to exclude DNA contamination in the PCR reagents and primer dimers (single measurement). The analysed melting curves did not show shoulder peaks or extra peaks, so that non-specific amplification reactions could be excluded. Furthermore, DNA samples with a known number of *T*. *urticae* specimens were carried in varying dilutions, which served as reference points. Fluorescence intensity over time was determined using the Ct value with QuantStudioTM 12K Flex Software v1.2. The standard curve of the logarithmic quantity was plotted against the corresponding Ct value to quantify the spider mites in the samples. Results were calculated in spider mite number/ g sample. All samples were tested in duplicate under the conditions of HRM real-time PCR assay.

Statistical analysis was completed with GraphPad Prism 8 Version 8.3.0 (538) (GraphPad Software, San Diego, CA, USA) based on Analysis of Variance (ANOVA) and linear regression. ANOVA was performed with not significant (ns) p > 0.05, and significant correlations at * p ≤ 0.05, ** p ≤ 0.01, **** p ≤ 0.0001. Normal distribution was verified by Quantile-Quantile-Plot. Linear regression analysis was performed after outliers were removed to determine if the initial spider mite concentration in the hotspot influenced the further development of spider mite abundance during the measurement period.

### HRM real-time PCR assay sensitivity and specificity

The HRM real-time PCR assay in this study was modified from Li et al. (2015) [22] without probes, therefore species specificity of the assay was tested *in silico*. DNA sequences of *Tetranychus* species listed in S4 Table from the GeneBank database were compared *in silico* [22, 24–33]. Unreliable sequences were excluded. The ITS1 sequences of the species *T*. *collyerae*, *T*. *desertorum*, *T*. *evansi*, *T*. *ezoensis*, *T*. *kanzawai*, *T*. *lambi*, *T*. *ludeni*, *T*. *macfarlanei*, *T*. *merganser*, *T*. *misumaiensis*, *T*. *neocaledonicus*, *T*. *okinawnus*, *T*. *pacificus*, *T*. *parakanzawai*, *T*. *phaselus*, *T*. *piercei*, *T*. *pueraicola*, *T*. *takafujii*, *T*. *truncatus*, and *T*. *turkestani* were analyzed. Primer alignment (primer BLAST) was used to determine the *in silico* resulting amplicons ranging from 138 to 155 bp in length. The multiple alignment program MAFFT and MUSCLE were used to identify indeels and SNPs in these amplicons. Melting profiles of the amplicon sequences of the *Tetranychus* species were determined via the software uMELT Quartz (Reaction conditions: free [Mg++]: 2.5 mM, [Mono+]: 20 mM, DMSO: 0%, Resolution: 0. 25°C) and uAnalyze^sm^ (Reaction conditions: free [Mg++]: 2.5 mM, [Mono+]: 20 mM, DMSO: 0%) and compared with the melting curve of *T*. *urticae* (Reference from GenBank accession number HM565874).

PCR sensitivity for target DNA detection was determined using a dilution series (up to 10^−4^) of extracted DNA from counted spider mites. Extracted DNA from a known number of spider mites was tested and detected against the dilution series.

Furthermore, it was important to test the PCR assay for possible inhibition by humic substances contained in the DNA samples obtained from soil and plant material. Even small amounts of these potent inhibitors can lead to complete failure of enzymatic reaction. DNA isolation in this study was performed using the NucleoSpin® Soil kit (Macherey-Nagel), specifically designed for soil and environmental samples to minimize humic substances. The kit manufacturer guarantees the complete removal of humic substances and other PCR inhibitors typically found in soil and sediment samples (as confirmed in our laboratory) (User’s Manual May 2016, Rev. 06). Nevertheless, aliquoted samples in which no fluorescent signal was detected were spiked with a known concentration of standard *T*. *urticae* DNA and analyzed by Ct value comparison to the standard. This procedure was performed with DNA extracted from plant material and soil samples.

## Results

### Performance, specificity and sensitivity of the HRM real-time PCR assay

**Fig 3:**
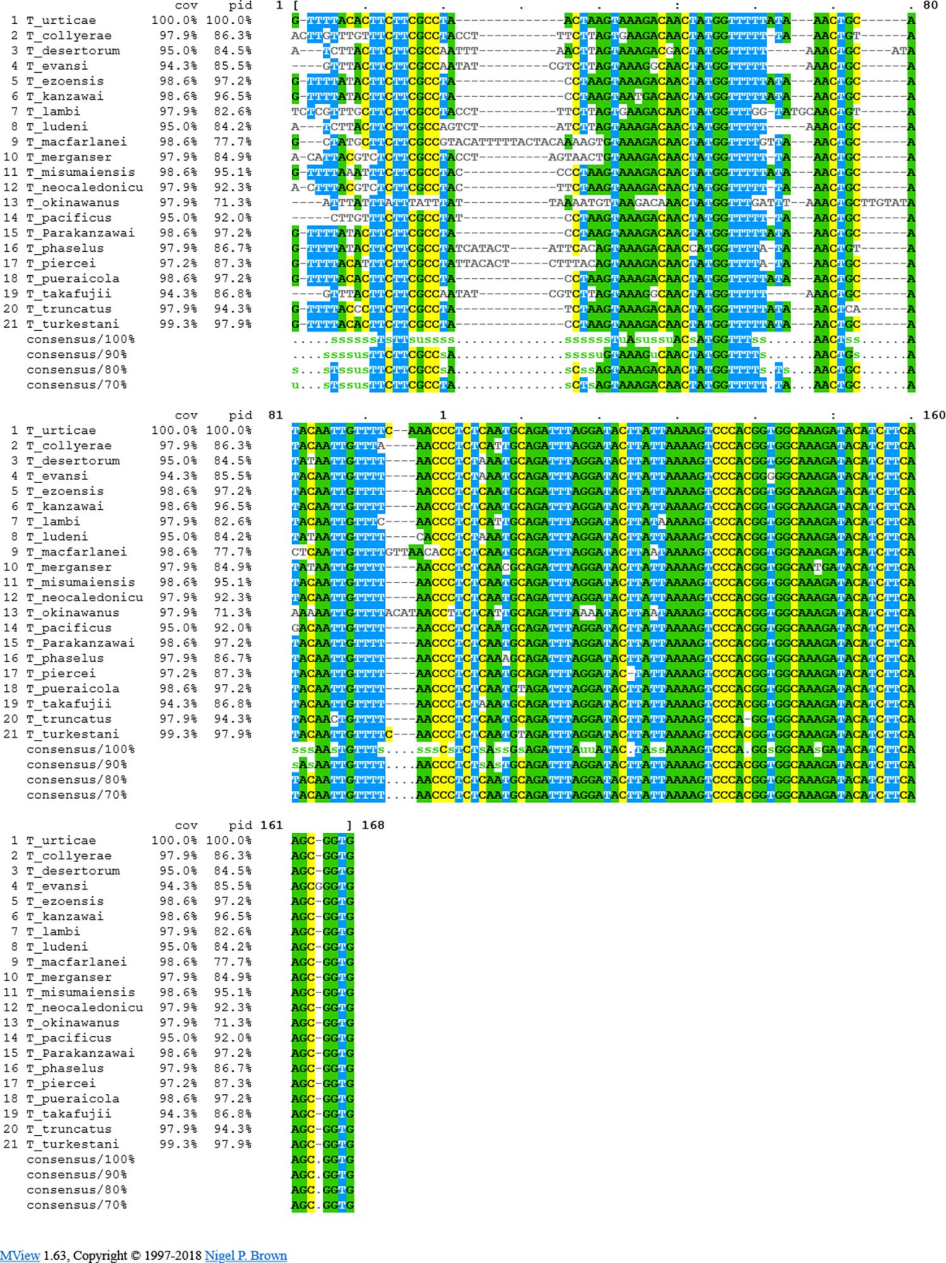
Alignment of partial amplicon sequence from ITS1 sequence of *Tetranychus* species. Color-coded bases indicate sequence similarity between amplicons. Bases without color or dashed lines indicate mismatches between sequences.

The standard series was prepared using extracted DNA from *T*. *urticae*. *T*. *urticae* was determined securely by the distributor (Katz Biotech AG, Baruth/Mark, Germany). There was no possibility to obtain other *Tetranychus* species, so alignment analyses were done *in silico*. Alignment comparisons of the entire sequence from forward to reverse primer of *Tetranychus* species showed that SNPs and indels are present in the amplicons. In the forward primer region, all *Tetranychus* species have SNPs, indels, or mismatches divergent from *T*. *urticae* (Fig 3). The species *T*. *truncatus*, *T*. *parakanzawai*, *T*. *kanzawai*, and *T*. *ezoensis* have two SNPs in the forward primer region. The two species *T*. *piercei* and *T*. *phaselus* each have one SNPs and one indel, but more than four mismatches in the forward primer region. *T*. *turkestani* and *T*. *pueraicola* each have a mismatch at the crucial 3′-end of the forward primer. The species *T*. *evansi*, *T*. *takafujii*, *T*. *ludeni*, *T*. *lambi*, *T*. *merganser*, *T*. *misumaiensis*, *T*. *neocaledonicus*, *T*. *pacificus*, *T*. *collyerae*, *T*. *okinawanus*, and *T*. *desertorum* have more substantial mismatches (≥ 4), especially at the 5’-end of the forward primer (Fig 3). Just the two species *T*. *evansi* and *T*. *merganser* each have a SNP in the region of the reverse primer.

In our experimental analyses, we determined a melting temperature (Tm) of 79.24°C for *T*. *urtic*. The melting curve for *T*. *urticae* showed only a single, narrow peak, with no shoulder or extra melting peaks. This is also shown by the in silico melt profiles for *T*. *urticae* and other related species (S1 Fig). Melting curve shifts due to sequence variation excluded therefore about 5 to 6% of DNA-samples. Such cases occurred only in soil, not in leaf samples. These samples were not considered further and were rejected.

The *in silico* melt profiles of the very closely related *Tetranychus* species differ from the melt profile of *T*. *urticae* in varying degrees of distinctness. A manual analysis of all samples compared to the standard is therefore necessary. The similarity of the melting profiles increases, the closer the degree of relationship of the species is, and thus the similarity of the amplicon sequences increases. Melting temperatures at the maximum fluorescence signal of the HRM real-time PCR (*in silico*) are listed in S5 Table. The individual melting profiles (*in silico*) of the most closely related *Tetranychus* species (*T*. *turkestani*, *T*. *truncatus*, *T*. *pueraicola*, *T*. *parakanzwai*, *T*. *kanzawai*, *T*. *ezoensis*) compared to *T*. *urticae* are shown in S1 Fig. The melting profiles often have the same melting temperature but a different maximum fluorescence signal.

PCR sensitivity for target DNA detection was determined using a dilution series of extracted DNA from counted spider mites. Dilution was up to 10^−4^ resulting in a sensitivity of 0.0077 spider mites/ g soil at a Ct > 40 cycles. The amplification efficiency is > 98% with a strong correlation coefficient (r^2^ = 0.99). Extracted DNA from a known number of spider mites was tested and detected against the dilution series (data not shown), providing experimental consistence.

During an overwintering analysis of spider mites in the soil, a large number of samples was noted in which the target DNA could not be amplified, so inhibition of PCR was suspected. For this reason, aliquoted samples in which no fluorescent signal was detected were spiked with a known concentration of standard *T*. *urticae* DNA and were analyzed. This procedure was performed with DNA extracted from plant material and soil samples. The *T*. *urticae* spiked plant material samples revealed a Ct value of 24.65 ±0.25 (n = 5), and the corresponding standard showed a Ct value of 24.52 ± 0.07. No inhibition occurred in the extracted DNA samples obtained from plant material of cucumber. The soil samples were spiked with a lower concentration of the standard DNA and had an average Ct value of 36.15 ±0.61 (n = 4), whereas the Ct value of the corresponding standard was 34.5 ±0.78, which is in the tolerable range with a deviation of 4.7%.

### Spider mite abundance in the soil 2017 to 2019

**Fig 4:**
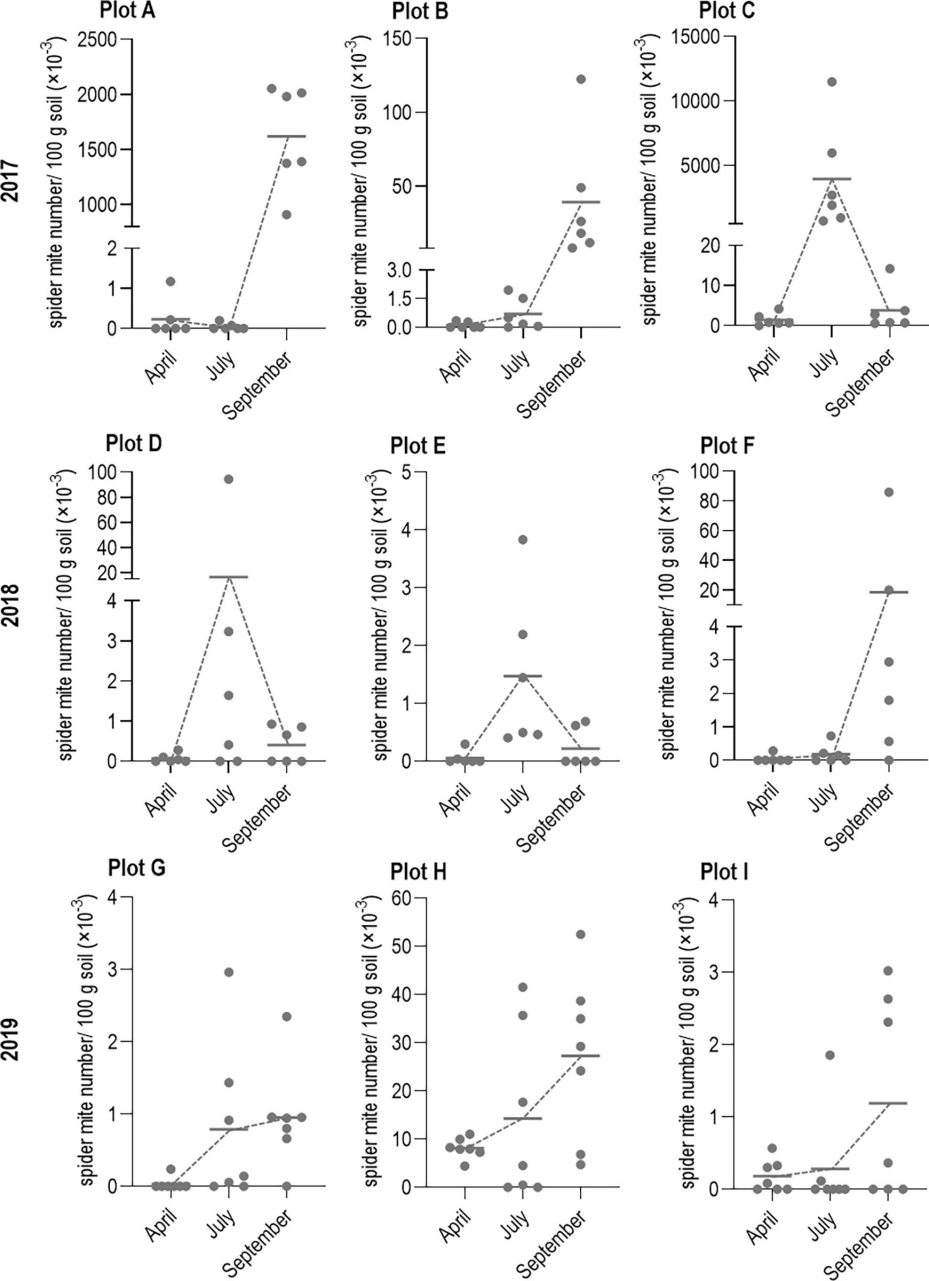
Spider mite abundance in the soil of cucumber plots measured 2017 to 2019. Three different open field cucumber plots were investigated per year: A-C 2017; D-F 2018; G-I 2019. Sampling always took place in April, July, and September. Spider mites were detected by HRM real-time PCR (spider mite number/ 100 g sample). A plotted point includes the mean of a sample transect of a trial plot consisting of eight mixed sample points (= 16 individual values). The seasonal development of the spider mite occurrence in the cucumber field is illustrated by a dotted line. The solid lines represent the mean values of all transects at the measurement times.

It was observed that spider mites were distributed very heterogeneously and occurred in hotspots within and between the trial plots (Fig 4). Fig 4 shows the seasonal abundance of spider mites in the soil. A plotted point in Fig 4 includes the mean of a sample transect of a trial plot consisting of eight mixed sample points (= 16 individual values). Spider mites could be detected in the soil in varying concentrations up to 11.48 spider mite number/ 100 g soil (Fig 4, highest average value of a transect in plot C). The average value of spider mite abundance per plot did not exceed 0.002 spider mites per 100 g of soil in three of nine plots examined (Fig 4, plot E; G; I) at any time. In the remaining six plots, the highest average value per plot was 1.592 spider mite number/ 100 g soil in plot A (September), 0.043 spider mite number/ 100 g soil plot B (September), 4.11 number/ 100 g soil in plot C (July), 0.0166 spider mite number/ 100 g soil in plot D (July), 0.0185 spider mite number/ 100 g soil in plot F (September) and 0.026 spider mite number/ 100 g soil in plot H (September). Thus, the most spider mites occurrence were measured in 2017, and the fewest in 2019. A seasonal increase in spider mite abundance was observed at all plots with the highest concentration in July (Fig 4, plots C; D; E) or twice as often in September (Fig 4, plots A; B; F; G; H; I). In April, without plants growing on the fields, spider mite infestations always averaged less than 0.001 spider mites per 100 g of soil, except for 2019 (Fig 4, plot H). If the maximum of spider mites was already reached in July, a distinct decrease of spider mites was registered in September.

### Spider mite abundance in the phyllosphere vs soil

**Fig 5:**
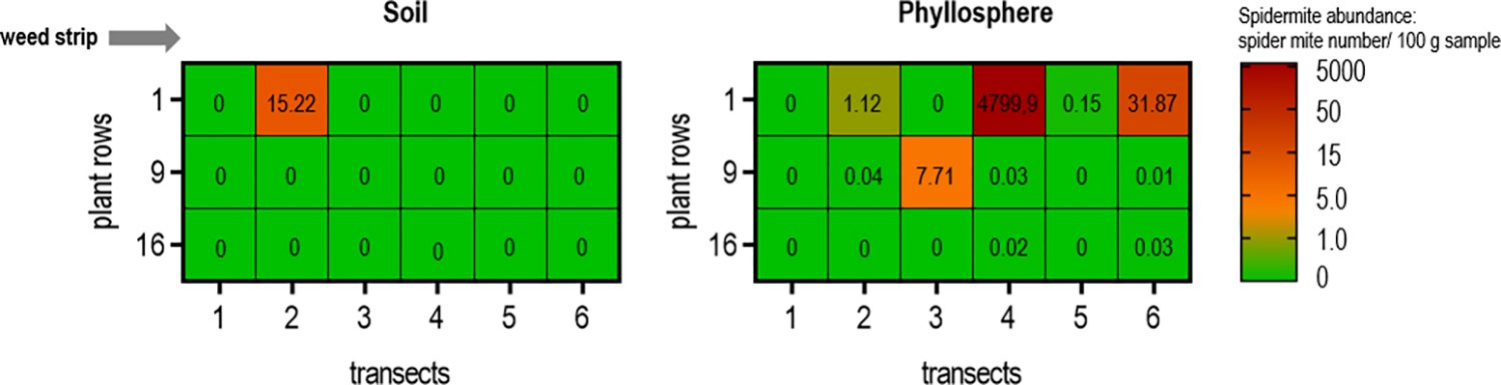
Spider mite abundance in the phyllosphere and soil of an open field cucumber cultivation. Plot dimensions were 500 m long and 30 m wide included 19 plants rows and 6 transects. Three to five leaves and two soil samples were collected at one sampling point and merged together for analysis. Quantification of spider mites in the samples was conducted by HRM real-time PCR (spider mite number/ 100 g sample). The spider mite number/ 100 g sample increases from green to red. Spider mite colonization of the crop is highest at the field edge adjacent to the weed strip. The spider mite colonization of soil and phyllosphere do not influence each other.

In 2018, additional sampling of soil and foliage was conducted at plot D in mid-June. The purpose was to determine spider mite infestations in the phyllosphere and soil and the relationship between them. High spider mite infestations on leaves compared to very low occurrences in the soil at the same sample point were distinct (Fig 5). Therefore, the colonization of leaves and soil with spider mites appears to be independent of each other. High spider mite abundance on leaves was recorded significantly more often on plot edges adjacent to the weed strip with highest value of 4,799.9 spider mite number/ 100 g leaves (transect 4, row 1). A increased spider mite occurrence on the leaves (7.71 spider mite number/ 100 g sample) was recorded at one sample point in the middle of the field (transect 3, row 9). Only one spider mite accumulation with 15.22 spider mite number/ 100 g sample was detected in the soil (transect 2, row 1) compared to spider mite abundance on leaves at the same sampling point with 1.12 spider mite number/ 100 g sample.

### Effect of predatory mites on spider mites in field-grown cucumbers

**Fig 6:**
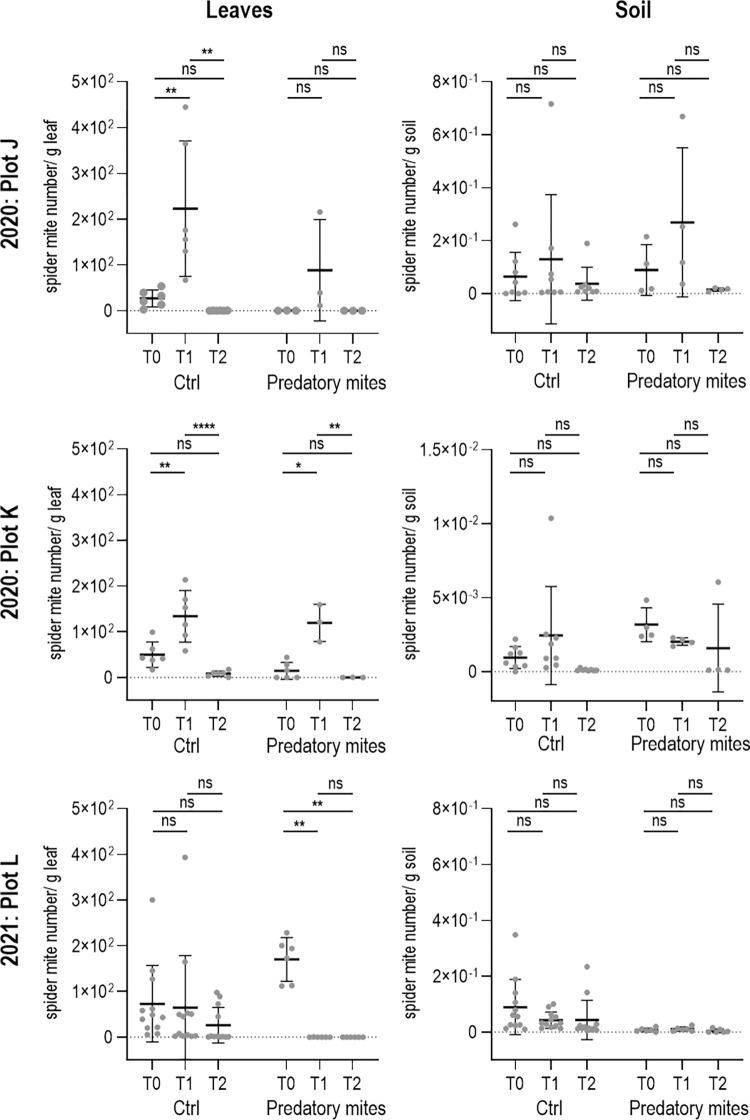
Effect of predatory mites on spider mite hotspots in cucumber cultivation in 2020 and 2021. Soil and leaf samples from spider mite hotspots located in cucumber fields were analysed weekly for three weeks during main cucumber harvest (first sampling time: T0; second sampling time: T1; third sampling time: T2). Untreated control spider mite hotspots (- predatory mites) were compared with hotspots where predatory mites were released (+ predatory mites). Predatory mites (*N*. *californicus*) were applied between T0 and T1. The control hotspots (-predatory mites) were therefore also colonized with spider mites and represented the natural infestation process without countermeasures. Predatory mites show a reducing effect on spider mites in the hotspot only when the initial level of spider mite colonization is high. Spider mites were detected by HRM real-time PCR. ANOVA was performed with not significant (ns) p > 0.05, and significant correlations at * p ≤ 0.05, ** p ≤ 0.01, **** p ≤ 0.0001. Shown are the mean values with standard deviation, dots represent individual measured values. Values for ANOVA of plot J; K; L are shown in S1–S3 Tables.

High spider mite growth dynamics and heterogeneity were observed on leaves and soil in the hotspots of all three plots during the measurement period (Fig 6). The leaves were 1,000 times more infested with spider mites compared to the soil.

In 2020, plots J and K differed significantly in the severity of their initial spider mite colonization on the leaves (0.358 and 29.258 spider mite number/ g sample), allowing the effect of predatory mite application to be estimated under different starting conditions. The control hotspots had initial spider mite concentrations of 27.026 (plot J) and 50.223 spider mite number/ g leaf (plot K). The peak spider mite densities on the leaves and in the soil were reached mostly in the second week of the measurement (T1) (Fig 6, plots J; K). Thereafter, spider mite occurrence on leaves and in the soil decreased considerably in both the controls and the hotspots with predatory mite application. Thus, similar spider mite abundance dynamics could be seen on the leaves and in the soil in all hotspots (except for plot K, predatory mites; with a generally very low spider mite abundance in the soil). The hotspots with predatory mite application exhibited the lowest spider mite occurrences (plot J (T2) 0.038 and plot K (T2) 0.307 spider mite number/ g sample) compared to the control (plot J (T2) 0.231 and plot K (T2) 8.470 spider mite number/ g sample) at the end of the measurements (T2).

In 2021, the maximum spider mite density was measured at the beginning of the analysis in the control (on leaves 73.282 spider mite number/ g; in soil 0.090 spider mite number/ g) and the hotspot with *N*. *californicus* release (on leaves 170.135 spider mite number/ g; in soil 0.008 spider mite number/ g) (Fig 6, plot L). The hotspot with predatory mite release showed a significantly greater spider mite decrease than the control at measurement times T1 and T2. There, the average of 170.135 detectable spider mite number/ g leaf at T0 reduced to approximately 0.090 spider mite number/ g leaf at T2, a reduction of almost 100%. In comparison, spider mite abundance in the untreated control hotspots drops to 26.284 by time T2, a reduction of 64.13%. The abundance of spider mites in the soil was generally low and reduced by approximately half at T2 in both the control and the hotspot with predatory mite application.

Linear regression analysis of spider mites on leaves showed no correlation between the initial spider mite occurrence in the hotspot and the ongoing spider mite dynamics during the measurement neither in the control nor in the treated hotspots (simple linear regression of control (T0:T1): F(1, 1) = 118.0, p = 0.0584; control (T0:T2): F(1, 1) = 9.625, p = 0.9185); predatory mite release (T0:T1): F(1, 1) = 5.208, p = 0.2629; predatory mite release (T0:T2) F(1, 1) = 0.08467, p = 0.8197). It was assumed that a particularly low initial spider mite concentration would continue to increase despite the release of predatory mites (due to the migration of predators as a result of the food deficiency).

## Discussion

### Real-time PCR assay

Morphological identification of tetranychid species is difficult due to limited diagnostic characters, small size and large phenotypic plasticity of key characters. Morphological identification requires expertise and rarer male individuals. However, the very closely related spider mites *T*. *turkestani* and *T*. *urticae*, for example, cannot be distinguished based on their morphology [22, 28, 34–36]. Molecular techniques such as mtDNA barcoding or ITS-RFLP were recommended for species identification of this genus [24, 29]. These approaches involve technical effort, many handling steps, extensive library of banding patterns and are time-consuming [37]. The HRM real-time PCR assay used here combines the annealing and extension steps of the reaction, making it much faster than conventional PCR or PCR-RFLP and allowing simultaneous analysis of a large number of samples without post-PCR processing. The real-time PCR assay is highly specific (detects all life stages of *T*. *urticae*), allows quantification of the spider mites and reduces the time required to approximately four hours (compared to PCR-RFLP with 8 hours). The combination of real-time PCR assay with the HRM method allows genotype-specific discrimination upon melting curve shift due to the intercalating fluorescent dye [23]. The HRM method further refined the real-time PCR assay and enabled the rejection of 5 to 6% false-positive samples each year via the melting curve shift.

### HRM real-time PCR assay specificity

The HRM real-time PCR assay in this study was modified from Li et al. (2015) [22] without probes, therefore species specificity of the assay was tested. The *in silico* mismatches, SNPs, and indels determined in the forward primer region in the related *Tetranychus* species compared to *T*. *urticae* suggest that successful primer annealing would occur only in *T*. *urticae*, primarily because mismatches can affect annealing temperature. The probability that amplification occurs decreases with increasing deviation from the primer sequence. In addition, it was shown by Lefever et al. (2013) [38] that at four mismatches or more in one of the primers, PCR is completely blocked, resulting in the majority of *Tetranychus* species not being amplified due to the primers used here. Species that cannot be amplified include *T*. *collyerae*, *T*. *desertorum*, *T*. *evansi*, *T*. *lambi*, *T*. *ludeni*, *T*. *macfarlanei*, *T*. *merganser*, *T*. *misumaiensis*, *T*. *neocaledonicus*, *T*. *okinawnus*, *T*. *pacificus*, *T*. *phaselus*, *T*. *piercei*, and *T*. *takafujii*. Annealing of the forward primer cannot be entirely excluded in very closely related species with only two SNPs (*T*. *parakanzawai*, *T*. *kanzawai*, *T*. *ezoensis*, *T*. *truncatus*) or only one SNP at the 3’-end (*T*. *turkestani*, *T*. *pueraicola*). However, Lefever et al. (2013) [38] also showed that especially mismatches at the 3’-terminus of a primer (as present in all very closely related *Tetranychus* species) have a severe impact on amplification due to steric hindrance, etc.. Therefore, we assume a low probability of amplification of *Tetranychus* species other than *T*. *urticae*. There is no evidence in the literature that the very closely related *Tetranychus* species have been detected in Germany, even though *T*. *turkestani* and *T*. *kanzawai* have already occurred in various European countries such as Greece, France, Spain, the Netherlands, and Poland (*T*. *pueraicola*, *T*. *truncatus*, *T*. *parakanzawai* and *T*. *ezoensis* only in the Asian region or the USA) [4]. The occurrence of these species closely related to *T*. *urticae* cannot be excluded in field cucumber cultivation in eastern Germany but seems unlikely according to current knowledge and thus their detection by HRM real-time PCR in the present study. In addition, the individual melting profiles of each sample were used to distinguish them. All analyzed species differed in their amplicon sequence by at least three bases. HRM real-time PCR allows the identification of individual SNPs. Sequence divergence of often 2 to 3% between the different *Tetranychus* species amplicons and thus a change in the melting profile make a high-resolution separation of the samples possible. Nevertheless, the method is critical in closely related species and reaches its limits. The *in silico* melting profiles of the amplicons of the *Tetranychus* species, which could be amplified, show a very similar shape and often the same melting temperatures at the maximum fluorescence signal in closely related species as in *T*. *urticae*, thus confusion could happen. If the amplicon sequences change due to additional SNPs or indels or their omission, the melting profile would also change, which could difficult evaluation or make it impossible to distinguish between samples, thus limiting the robustness of the method. Especially the species *T*. *turkestani*, *T*. *truncatus*, *T*. *pueraicola*, *T*. *parakanzawai*, *T*. *kanzawai* and *T*. *ezoensis* show a high degree of melt profile similarity with *T*. *urticae*. *In silico*, the melting profiles of the related species often showed the same Tm but different fluorescence signals. In practice, however, a weaker fluorescence signal may also indicate inhibitors or samples with low expression levels. A verified *T*. *urticae* standard must be carried at each HRM real-time PCR. Verification of primer annealing and possible amplification in combination with melt profile analysis must be performed with morphologically determined and sequenced specimens of the *Tetranychus* species. The broad detection approach is suitable for monitoring the pest *T*. *urtiace* and the closely related species and their invasion and overwintering.

The ITS1 region of *Tetranychus* species (target in this study) is less conserved than the ITS2 region and exhibits 2 to 3% nucleotide divergence in *T*. *urticae* exemplars collected in different countries (Europe, USA, Japan) at a total length of 450 bp according to Hurtado et al. (2008) [25] Nucleotide divergence would potentially lead to a change in the melting profile. The primer pair used in this study results in an amplicon of 141 bp when amplified. The probability of nucleotide divergence decreases with a smaller base pair number, even if possible. The amplicon sequences available in GenBank (S4 Table) were aligned against each other using BLAST to detect possible variations within the amplicon. A divergence was detected only in the sequences submitted in GenBank under accession numbers HM565880 and HM565883, with no change in Tm and melting curve profile (base swipe). The *T*. *urtiace* sequences available in Genbank originate from specimens collected in different countries, and therefore sequence divergence is more likely.

### Distribution and seasonal abundance of spider mites in cucumber fields

Due to rare knowledge about the dynamics and occurrence of spider mites in soil, it is difficult to assess whether soil serves as a habitat, niche or shelter for spider mites and thus as a source of the infestation in open field cucumbers. The purpose of this analysis was to estimate how intensively the soil must be involved in the spider mite control and when the highest potential infestation pressure comes from the soil. Especially in the Spreewald region, which is characterized by intensive cucumber cultivation and its geographically protected cucumber brand, possible cucumber cultivation areas are limited, and crop rotation (if possible at all) is closely timed. The cucumber plants are not removed from the fields at the end of the season but plowed into the soil. With this background, our studies on the abundance of spider mites in the soil concerning overwintering rate and emergence during the season are of particular interest, especially in cucumber cultivation. Other studies have also shown that spider mites prefer to overwinter in dark places and were detectable in clods of soil [21].

The results show that few, often only traces, of spider mites were present in the soil of open field cucumbers. The spider mites were concentrated mainly in hotspots heterogeneously spread in the field. This type of distribution and occurrence was proven over three years in nine different open field cucumber plots. Only in one trial plot, spider mites were detected slightly elevated in about one third of the total number of samples. Minor spider mite occurrences distributed in individual samples were also detected in soil and litter from cotton fields in Australia [17] or in apple orchards in South Korea [39].

Despite the low occurrence of spider mites in the soil, seasonal spider mite dynamics could be detected. The seasonal abundance of the spider mites in the soil of cucumber fields showed three main phases: first, an initial very weak colonization at seedling emergence, second, a mid- to late-season increase with varying intensity, and third, a decline in colonization. Spider mite abundance in the soil was particularly low to absent in April, indicating that overwintering or prolonged residence of spider mites in the field soil is minor. The bare unprotected soil, as well as the habitat destruction of overwintering spider mites in soil and litter between growing seasons due to abrasion, burial, and climatic conditions, are probable causes of mortality [18]. Probably, there is no infestation pressure from spider mites living in the soil at the time when the cucumber seedlings emerge. However, a general increase in the spider mite incidence of the soil was observed during the cucumber growing season with peaks in July or September on all plots of the three years of the analysis. This may have been the result of the effects of cultivation. Open field cucumbers are grown with fleece cover from mid-April or without cover from mid-May to the end of September in temperate latitudes. The relatively late cultivation of cucumber plants in the field provides a potential fresh food source for spider mites, especially during the dry summer months, while surrounding plants already have lost food attractiveness due to pest, climatic damage and senescence of the plants. In our study, we observed that cucumber plant colonization occurred primarily from the edge of the field adjacent to the weed strip indicates that a substantial portion of the colonizing spider mite population enters the crop from an external source. This so-called edge effect was also found in other studies investigating spider mite colonization in cotton fields [17]. The edge effect in our study concentrated on the infestation of the plants since the sometimes very high spider mite infestation on the cucumber plants did not correlate with the infestation density in the soil. Spider mite hotspots within the field can also be attributed to wind dispersal (or crawling) of the mites rather than infestation due to soil colonization [40]. Our study shows that the abundance of spider mites in soil was mainly very low and that there was no clear evidence of economic relevance from soil-colonizing spider mites. In addition, spider mite hotspots in the soil did not appear to increase spider mite infestations on the plants and vice versa (Fig 4). There seemed to be no supporting infestation effect or "storage" of spider mites in the soil. Consequently, the soil seems to be occupied only temporarily (e.g. by crawling even across the soil [35]) and not as a permanent habitat or shelter by spider mites. As previously reported in other studies, weed strip management appears to be a necessary measure in successful spider mite control [17, 41, 42].

### Predatory mites release at spider mite hotspots

The biological control of *T*. *urticae* with predatory mites seems to be an alternative to chemical control agents due to the frequent resistance of *T*. *urticae* to such agents. The application of predatory mites is already well-known and successfully used in the greenhouse [15, 43, 44], whereas field application has been little researched and appears uneconomical for wide-scale release due to high costs [15, 45]. Possible natural occurrences of *N*. *californicus* in the climatic latitudes of the Spreewald gherkin cultivation region are most likely minor and should not influence the study. In addition, there were no greenhouses or other fields with *N*. *californicus* utilization in the vicinity of the surveyed croplands from where the predators could have dispersed. To date, natural populations of *N*. *californicus* outside the greenhouse have been recorded only in Asia, South America, southern North America, parts of southern Europe, and along the Mediterranean Sea [46, 47]. Sustained establishment and applied biological control in the environment rely on the ability of predators to adapt to the local environment of the region in which they are released. For example, *N*. *californicus* naturally occurs considerably less frequently in the cooler north and warmer south of Japan than in central Japan. [46] In Central and Northern Europe including Germany and the cultivation area of Spreewald gherkins natural occurrences of this predator have not been documented so far [48, 49]. A study in the UK estimates that up to 7 generations of a commercially available *N*. *californicus* can theoretically develop there within a year in the field, but overwintering mite abundance is low and dependent on diapausing ability [14]. In Korea, on the other hand, *N*. *californicus* was found to survive exposed to bare soil for no longer than 27 days, and no overwintering of mites occurs despite shelter [50].

Our objective was to capture the effect of a targeted single release of *N*. *californicus* into spider mite hotspots within the cucumber field, thus limiting the application of predators to a small area. One more objective was to conduct the investigations in an environment as natural as possible without manipulations. This means that all spider mite hotspots were of natural origin with natural variances. Furthermore, we ensured that the application of the predatory mites is practicable and applicable for the growers. The single release of predatory mites occurred after the level of spider mite infestation was determined in the visually detectable hotspot.

### Trial plots 2020

In 2020, the trial plots had an average initial spider mite concentration of already 26.716 spider mite number/ g sample. Nevertheless, spider mite infestation continued to increase sharply in the hotspots with released predatory mites and without release until the second measurement indicating a too late release of predatory mites or an inappropriate predator-prey ratio, as found in strawberry and tomato fields [8, 15]. By the last measurement, the spider mite population collapsed in both hotspot variants. A possible reason could be the migration of the *T*. *urticae* due to the decreasing food quality in the hotspots [17].

### Trial plot 2021

In 2021, spider mite infestation was significantly reduced after predatory mites release. The release of predatory mites as a control agent seems to break the spider mite dynamics and significantly reduce them permanently. The different initial levels of spider mite colonization in the hotspots studied in 2020 and 2021 were conspicuous. Although *N*. *californicus* is robust to food deficiency [13, 51, 52], this predator operates more effectively when a high level of *T*. *urticae* is initially present [15]. Even we did not observe any influence of the initial spider mite concentration on the ongoing spider mite occurrence on the leaves, we observed a severe reducing and persistent effect of the predatory mites at initially high spider mite concentrations in the hotspot. More extensive studies with different initial spider mite concentrations in hotspots are needed to determine this with confidence.

The spider mite infestations in hotspots with predatory mites release were always below the infestation level of the control hotspots in all plots at the end of the measurement series, indicating a possible control effect of the predators against spider mites. Similar success with predatory mites was also seen in field trials with blackberries, strawberries and hops [15, 16, 43]. Akyazi and Liburd (2019) even showed a permanent reduction of *T*. *urticae* infestation on blackberries from the second week after *N*. *californicus* application [43]. Thus, further application was not necessary and consequently, costs can economize. In addition, predatory mites leave odorants on plants that have a deterrent effect on spider mites, which avoid these plants [53]. Various factors such as the predatory mites themselves, their odorants, declining food quality of the plants, or other biotic and abiotic influences can contribute to the reduction of spider mite abundance in the hotspots. Therefore, it would be relevant for future research to determine how the spider mite ratio changes around the hotspot to find out if spider mite numbers are actually decreasing or if they are only migrating. Studies show that an initial predator: prey ratio of 1:10 is necessary for successful control of phytophagous mites so that a single application of *N*. *californicus* results in tolerable levels of *T*. *urticae* throughout the season [8, 15, 54]. The individual adaptation of the predator: prey ratio for each hotspot would only be possible with extensive monitoring (e.g. by real-time PCR) or at different application times and appears impractical. Alternatively, an additional application of a highly specific predator such as *P*. *persimilis* may be used to compensate the low prey specificity of *N*. *californicus* and to increase the spider mite control [52, 55, 56].

## Conclusions

We examined the seasonal abundance and distribution of spider mites in the soil of open field cucumber cultivation and found that the occurrence of spider mites in this habitat was very low. With this extensive study, soil can be ruled out as a habitat for spider mites, and attention to spider mite pest control in cucumber production can be directed to plant infestations. Thus, our study provides another clue to the lifestyle of spider mites and a better understanding of spider mite control for practitioners. The spider mite control should focus on weed strips and spider mite hotspots in the field. A single release of predatory mites as a biological control agent may contribute to a localized reduction in spider mite infestations, although this is unlikely to be sufficient as a stand-alone measure. Localized control limited to hotspots enables precise monitoring and cost control. This study provides the basis for further research on the single release of predatory mites in spider mite hotspots with varying levels of infestation. In this context, the HRM real-time PCR assay provides a reliable and rapid detection method for spider mites. For the final validation of this method for quantifying a spider mite infestation in the field, a comparative count of spider mites of all ontogenetic stages should be conducted.

## Supporting information

S1 Table Ordinary one-way ANOVA to the data of Fig 6, plot K—Multiple comparison. Listed are the p-values and significance level of ANOVA to plot K. Spider mite (*T*. *urticae*) occurrences on leaves and in soil in untreated spider mite hotspots (Crtl = control) and spider mite hotspots treated with *N*. *californicus* (N. cal.) were examined. Investigations were conducted at the time points T0, T1 and T2, one week apart. The predatory mites were applied between T0 and T1.
(DOCX)

S2 Table Ordinary one-way ANOVA to the data of Fig 6, plot J—Multiple comparison. Listed are the p-values and significance level of ANOVA to plot J. Spider mite (*T*. *urticae)* occurrences on leaves and in soil in untreated spider mite hotspots (Crtl = control) and spider mite hotspots treated with *N*. *californicus* (N. cal.) were examined. Investigations were conducted at the time points T0, T1 and T2, one week apart. The predatory mites were applied between T0 and T1.
(DOCX)

S3 Table Ordinary one-way ANOVA to the data of Fig 6, plot L—Multiple comparison. Listed are the p-values and significance level of ANOVA to plot L. Spider mite (*T*. *urticae*) occurrences on leaves and in soil in untreated spider mite hotspots (Crtl = control) and spider mite hotspots treated with *N*. *californicus* (N. cal.) were examined. Investigations were conducted at the time points T0, T1 and T2, one week apart. The predatory mites were applied between T0 and T1.
(DOCX)

S4 Table ITS1 sequences of *Tetranychus* species for alignment analysis. (DOCX)

S5 Table ITS sequences of *Tetranychus* species for analysis of alignment, melt temperature (Tm) and maximum fluorescence signal of HRM real-time PCR. The specimens listed here with the corresponding accession number were used for the alignment (Fig 3) and the melting curves (S1 Fig).
(DOCX)

S1 Fig Melting profiles of closely related *Tetranychus* species compared with *T*.*urticae*. The profiles were plotted with uAnalyze^sm^ (Reaction conditions: free [Mg++]: 2.5 mM, [Mono+]: 20 mM, DMSO: 0%).
(TIF)
