# Supplementary material for: Determination of spider mite abundance in soil of field-grown cucumbers and in plants under predatory mite pressure in invasive infestations using HRM real-time PCR assay
Source: PLoS One. 2022 Jul 14;17(7):e0270068. doi: 10.1371/journal.pone.0270068 (PMC9282461; doi:10.1371/journal.pone.0270068)
Supplement: S2 Table — Listed are the p-values and significance level of ANOVA to plot J. Spider mite (T. urticae) occurrences on leaves and in soil in untreated spider mite hotspots (Crtl = control) and spider mite hotspots treated with N. californicus (N. cal.) were examined. Investigations were conducted at the time points T0, T1 and T2, one week apart. The predatory mites were applied between T0 and T1. (DOCX) [file pone.0270068.s002.docx]

**Supplement: Ordinary one-way ANOVA to the data of Figure 4.**

**S2 Table.** **Ordinary one-way ANOVA to the data of Figure 6, plot J - Multiple comparison.** Listed are the p-values and significance level of ANOVA to plot J. Spider mite (*T. urticae)* occurrences on leaves and in soil in untreated spider mite hotspots (Crtl = control) and spider mite hotspots treated with *N. californicus* (N. cal.) were examined. Investigations were conducted at the time points T0, T1 and T2, one week apart. The predatory mites were applied between T0 and T1.

| **2020 Plot J : Leaf** | | |
| --- | --- | --- |
| Tukey's multiple comparisons test | Summary | Adjusted P Value |
| Ctrl Leaf T0 vs. Ctrl Leaf T1 | ** | 0,0044 |
| Ctrl Leaf T0 vs. Crtl Leaf T2 | ns | 0,9915 |
| Ctrl Leaf T1 vs. Crtl Leaf T2 | ** | 0,0012 |
| N. cal. Leaf T0 vs. N. cal. Leaf T1 | ns | 0,7574 |
| N. cal. Leaf T0 vs. N. cal. Leaf T2 | ns | >0,9999 |
| N. cal. Leaf T1 vs. N. cal. Leaf T2 | ns | 0,7548 |
| **2020 Plot J : Soil** | | |
| Ctrl soil T0 vs. Ctrl soil T1 | ns | 0,9625 |
| Ctrl soil T0 vs. Crtl soil T2 | ns | 0,9993 |
| Ctrl soil T1 vs. Crtl soil T2 | ns | 0,9956 |
| N. cal. soil T0 vs. N. cal. soil T1 | ns | 0,6120 |
| N. cal. soil T0 vs. N. cal. soil T2 | ns | 0,9854 |
| N. cal. soil T1 vs. N. cal. soil T2 | ns | 0,2480 |
