# Supplementary material for: Determination of spider mite abundance in soil of field-grown cucumbers and in plants under predatory mite pressure in invasive infestations using HRM real-time PCR assay
Source: PLoS One. 2022 Jul 14;17(7):e0270068. doi: 10.1371/journal.pone.0270068 (PMC9282461; doi:10.1371/journal.pone.0270068)
Supplement: S4 Table — (DOCX) [file pone.0270068.s004.docx]

**Supplement: Tetranychus species, section Methodes**

**S4 Table. ITS sequences of *Tetranychus* species for alignment analysis.**

| **Species** | **ACC#** | **Origin** | **Reference** |
| --- | --- | --- | --- |
| *T. urticae* | HM565885 | Spain: ES, Canary, Granadilla de Abona | [24] |
| *T. urticae* | HM565878 | France: FR, La Gaude-Bord du Var | [24] |
| *T. urticae* | HM565886 | Spain: ES, Canary, Arico | [24] |
| *T. urticae* | HM565887 | Spain: ES, Canary, Arico | [24] |
| *T. urticae* | HM565879 | France: FR, Nice | [24] |
| *T. urticae* | HM565888 | Spain: ES, Canary, Aguimes | [24] |
| *T. urticae* | HM565875 | France: FR, Perpignan | [24] |
| *T. urticae* | HM565889 | Greece: GR, Ieraptera | [24] |
| *T. urticae* | HM565877 | France: FR, Saint Nazaire | [24] |
| *T. urticae* | HM565880 | Spain: ES, Vilademuls/Galliners | [24] |
| *T. urticae* | HM565881 | Spain: ES, Bordils/Juia | [24] |
| *T. urticae* | HM565883 | Spain: ES, S. Andreu de Llavaneres | [24] |
| *T. urticae* | HM565874 | France: FR, Elne | [24] |
| *T. urticae* | HM565876 | Spain: SP, S.Susanna/Cami de la Riera | [24] |
| *T. urticae* | HM565884 | Spain: ES, Canary, Santiago del Teide | [24] |
| *T. urticae* | HM565882 | Spain: ES, Bordils/Juia | [24] |
| *T. urticae* | HM565873 | France: FR, Elne | [24] |
| *T. urticae* | KP744522 | Japan | [22] |
| *T. urticae* | KP744523 | Japan | [22] |
| *T. urticae* | KP744525 | Japan | [22] |
| *T. urticae* | KP744530 | Japan | [22] |
| *T. urticae* | KP744531 | Japan | [22] |
| *T. urticae* | AM408035 | Unknown | [25] |
| *T. urticae* | AB076369 | Japan: Wakayama, Gobo | [26] |
| *T. urticae* | AB736033 | Japan: Hokkaido | [27] |
| *T. urticae* | AB736034 | Japan: Ibaraki | [27] |
| *T. urticae* | AB736035 | Japan: Okinawa | [27] |
| *T. urticae* | AB736036 | Japan: Nagano | [27] |
| *T. urticae* | AB736037 | Japan: Kanagawa | [27] |
| *T. urticae* | AB736038 | Japan: Niigata | [27] |
| *T. urticae* | AB738746 | Uganda | [28] |
| *T. urticae* | AB738747 | Spain | [28] |
| *T. urticae* | KF544954 | China: Nanjing | [29] |
| *T. urticae* | KF544955 | China: Shihezi | [29] |
| *T. collyerae* | KP744532 | New Zealand | [22] |
| *T. desertorum* | MW682844 | USA | [33] |
| *T. evansi* | AB735996 | Japan: Tokyo | [27] |
| *T. evansi* | AB735997 | Japan: Kagoshima | [27] |
| *T. evansi* | AB738755 | Japan: Chiba, Narita | [28] |
| *T. evansi* | FJ440673 | Brazil: Piracicaba | [30] |
| *T. evansi* | GU145105 | Argentina | [31] |
| *T. ezoensis* | AB735999 | Japan: Ibaraki | [27] |
| *T. ezoensis* | AB735998 | Japan: Iwate | [27] |
| *T. kanzawai* | AB076370 | Japan: Shizuoka, Kanaya | [26] |
| *T. kanzawai* | AB736007 | Japan: Tokyo | [26] |
| *T. kanzawai* | AB736003 | Japan: Okinawa | [26] |
| *T. kanzawai* | AB736000 | Japan: Shizuoka | [26] |
| *T. kanzawai* | AB736006 | Japan: Hokkaido | [26] |
| *T. kanzawai* | AB736001 | Japan: Ibaraki | [26] |
| *T. lambi* | KP744533 | Unknown | [22] |
| *T. ludeni* | AB736009 | Japan: Okinawa | [27] |
| *T. ludeni* | AB736008 | Japan: Ibaraki | [27] |
| *T. ludeni* | AB736010 | Japan: Chiba | [27] |
| *T. ludeni* | AB738754 | Colombia | [28] |
| *T. macfarlanei* | AB738756 | Philippines | [28] |
| *T. merganser* | AB738753 | Mexico | [28] |
| *T. misumaiensis* | AB736011 | Japan: Hokkaido | [27] |
| *T. neocaledonicus* | AB736013 | Japan: Okinawa | [27] |
| *T. neocaledonicus* | AB736014 | Japan: Okinawa | [27] |
| *T. neocaledonicus* | AB736012 | Japan: Tokyo | [27] |
| *T. neocaledonicus* | AB738752 | Japan: Tokyo | [27] |
| *T. okinawnus* | AB736015 | Japan: Okinawa | [27] |
| *T. pacificus* | AB738744 | USA | [28] |
| *T. parakanzawai* | AB736020 | Japan: Okinawa | [27] |
| *T. parakanzawai* | AB736018 | Japan: Okinawa | [27] |
| *T. parakanzawai* | AB736017 | Japan: Ibaraki | [27] |
| *T. parakanzawai* | AB736019 | Japan: Hokkaido | [27] |
| *T. parakanzawai* | AB736022 | Japan: Chiba | [27] |
| *T. parakanzawai* | AB736021 | Japan: Okinawa | [27] |
| *T. phaselus* | AB736023 | Japan: Ibarak | [27] |
| *T. phaselus* | AB736024 | Japan: Okinawa | [27] |
| *T. phaselus* | AB738751 | Japan: Kanagawa, Yokohama | [28] |
| *T. piercei* | AB736025 | Japan: Okinaw | [27] |
| *T. piercei* | AB736026 | Japan: Okinaw | [27] |
| *T. piercei* | AB736027 | Japan: Okinaw | [27] |
| *T. pueraicola* | AB076372 | Japan: Ibaraki, Hitachiohta | [26] |
| *T. pueraicola* | AB736028 | Japan: Ibaraki | [27] |
| *T. pueraicola* | AB736030 | Japan: Nara | [27] |
| *T. pueraicola* | AB736029 | Japan: Nagano | [27] |
| *T. takafujii* | AB257746 | Japan | [22] |
| *T. truncatus* | JN018058 | Bangladesh | [32] |
| *T. truncatus* | JN018057 | Bangladesh | [32] |
| *T. truncatus* | AB736032 | Japan: Kyoto | [27] |
| *T. truncatus* | AB736031 | Japan: Kyoto | [27] |
| *T. turkestani* | AM408032 | Unknown | [25] |
| *T. turkestani* | AB738745 | USA | [28] |
