# Supplementary material for: Determination of spider mite abundance in soil of field-grown cucumbers and in plants under predatory mite pressure in invasive infestations using HRM real-time PCR assay
Source: PLoS One. 2022 Jul 14;17(7):e0270068. doi: 10.1371/journal.pone.0270068 (PMC9282461; doi:10.1371/journal.pone.0270068)
Supplement: S5 Table — The specimens listed here with the corresponding accession number were used for the alignment (Fig 3) and the melting curves (S1 Fig). (DOCX) [file pone.0270068.s005.docx]

**Supplement: *Tetranychus* species with Tm, section Methodes**

**S5 Table.** **ITS sequences of *Tetranychus* species for analysis of alignment, melt temperature (Tm) and maximum fluorescence signal of HRM real-time PCR.** The specimens listed here with the corresponding accession number were used for the alignment (Fig 3) and the melting curves (S5 Figure).

| **Species** | **ACC#** | **Length [bp]** | **Origin** | **Tm [°C]** | **-dF/ dT** | **Reference** |
| --- | --- | --- | --- | --- | --- | --- |
| *T. collyerae* | KP744532 | 578 | New Zealand | 84.25 | 78.572 | [22] |
| *T. desertorum* | MW682844 | 1044 | USA | 83.5 | 79.816 | [33] |
| *T. evansi* | AM408033 | 1218 | Unknown | 84.75 | 78.056 | [25] |
| *T. ezoensis* | AB735998 | 818 | Japan | 84.25 | 75.368 | [27] |
| *T. kanzawai* | AB736006 | 818 | Japan | 84.25 | 78.068 | [27] |
| *T. lambi* | AB738743 | 1156 | New Zealand | 85.5 | 85.896 | [22] |
| *T. ludeni* | KP744529 | 1165 | Japan | 84 | 80.512 | [22] |
| *T. macfarlanei* | AB738756 | 1225 | Philippines | 84.75 | 94.18 | [28] |
| *T. merganser* | AB738753 | 1181 | Mexico | 85.0 | 81.732 | [28] |
| *T. misumaiensis* | AB736011 | 822 | Japan | 84.5 | 73.996 | [27] |
| *T. neocaledonicus* | AB738752 | 1163 | Japan | 84.5 | 82.44 | [28] |
| *T. okinawnus* | AB736015 | 912 | Japan | 81.75 | 58.312 | [27] |
| *T. pacificus* | AB738744 | 1160 | USA | 84.25 | 76.344 | [28] |
| *T. parakanzawai* | AB736019 | 818 | Japan | 84.25 | 75.368 | [27] |
| *T. phaselus* | AB738751 | 1171 | Japan | 84.0 | 80.204 | [28] |
| *T. piercei* | AB738750 | 1178 | Malaysia | 84.25 | 79.104 | [28] |
| *T. pueraicola* | KP744528 | 1175 | Japan | 84.0 | 78.248 | [22] |
| *T. takafujii* | AB257746 | 1162 | Japan | 84.5 | 77.78 | [26] |
| *T. truncatus* | JN018057 | 1197 | Bangladesh | 84.0 | 81 | [32] |
| *T. turkestani* | AM408032 | 1223 | Unknown | 84.25 | 80.334 | [25] |
| 1. *urticae* | HM565874 | 1205 | France | 84.0 | 78.32 | [24] |
