# Supplementary figures and images for: Determination of spider mite abundance in soil of field-grown cucumbers and in plants under predatory mite pressure in invasive infestations using HRM real-time PCR assay
Source: PLoS One. 2022 Jul 14;17(7):e0270068. doi: 10.1371/journal.pone.0270068 (PMC9282461; doi:10.1371/journal.pone.0270068)

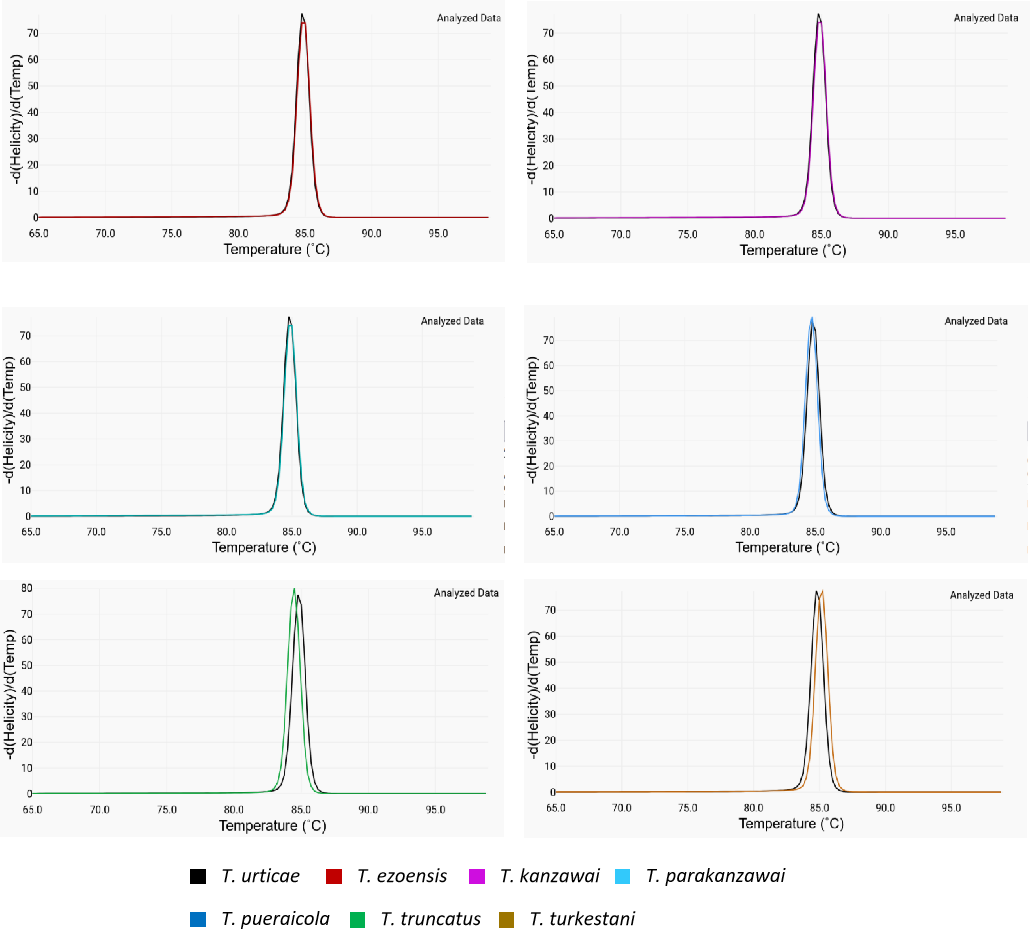

Supplement: S1 Fig — The profiles were plotted with uAnalyzesm (Reaction conditions: free [Mg++]: 2.5 mM, [Mono+]: 20 mM, DMSO: 0%). (TIF) [file pone.0270068.s006.tif]
